# Cultural suitability of schema therapy: a qualitative exploration of clinician views

**DOI:** 10.1080/00049530.2024.2412012

**Published:** 2024-10-10

**Authors:** Irene R. Martin, Sandra E. Stewart, Phillip Tchernegovski, Bethany D. Devenish

**Affiliations:** a School of Educational Psychology and Counselling, Faculty of Education, Monash University, Clayton, Australia; b Monash Krongold Clinic, Faculty of Education, Monash University, Clayton, Australia

**Keywords:** Schema therapy, reflexive thematic analysis, cultural adaptation, cultural competence

## Abstract

**Objective:**

There is a growing popularity in the worldwide use of schema therapy (ST) to address a variety of psychological disorders. Yet, research into the cultural suitability of ST is scarce. This study aimed to explore ST clinicians’ experiences of the cultural suitability of ST.

**Method:**

Eleven clinicians from nine countries were interviewed about their experiences of practising ST. Interviews were analysed using reflexive thematic analysis.

**Results:**

Two main themes, each with subthemes, were identified. The first theme was considerations for Cultural Context and Content: (i) Incongruence with Cultural Norms of Emotional Expression, (ii) “Maladaptive” or “Adaptive”, But in What Context?, and (iii) Issues Related to Familism when Addressing Parent/Critic Modes. The second theme focused on clinicians’ Cultural Competence: (i) Perceptions of Reduced Confidence and Competence in Providing Culturally Responsive Practice, and (ii) Clinicians’ Cultural Values Impacting ST Delivery. Participants discussed strategies to improve the cultural-responsiveness of ST, suggesting areas for further development.

**Conclusion:**

Present findings suggest that ST is not a one-size-fits-all approach, underscoring the need to improve the cultural responsiveness of ST, while better supporting clinicians to develop their cultural competence. Future research is needed to establish evidence-based cultural adaptations for ST.

## Key Points

**What is already known about this topic:**

1. Schema therapy (ST) is an evidence-based intervention gaining worldwide popularity with currently limited research investigating the cultural suitability of ST.
2. Mao et al. (2022) were the first to explore the acceptability of ST in a non-Western context. They found that although core concepts were generally accepted among schema therapists in Hong Kong and Singapore, substantial adaptations were required when implementing ST in Asian cultural contexts.
3. The 4-Domain Cultural Adaptation Model (CAM4; Sorenson & Harrell, 2021) outlines four domains that can be used to guide adaptation efforts for psychological interventions: (i) development and equivalence processes, (ii) cultural context and content, (iii) engagement efforts, and (iv) cultural competence.

**What this topic adds:**

1. ST clinicians endorsed the benefits of ST but reported that adaptations were required to improve the cultural suitability of ST.
2. Participants reported incongruence between standard ST and some cultural values including: (i) Incongruence with Cultural Norms of Emotional Expression, (ii) “Maladaptive” or “Adaptive”, But in What Context?, and (iii) Issues Related to Familism when Addressing Parent/Critic Modes. Participants also reflected on their own cultural competence in ST including: (i) Perceptions of Reduced Confidence and Competence in Providing Culturally Responsive Practice, and (ii) Clinicians’ Cultural Values Impacting ST Delivery.
3. ST is not a one-size-fits-all approach and may require adaptations to techniques (e.g. Imagery Rescripting and Limited Reparenting) and the therapeutic approach when applied with diverse clients to improve its cultural suitability. Clinicians must also be better supported to practice culturally responsive ST through access to appropriate professional development opportunities (e.g. supervision and training).

In increasingly globalised societies, cultural responsiveness in mental health delivery is an ethical imperative (Parsonson, 2021; Ridley et al., 2021). Culturally responsive practice includes an awareness of cultural factors that may influence the therapeutic process and the meaningful inclusion and consideration of culture in therapeutic approaches and techniques to better meet the needs of diverse clients (Benuto et al., 2021; Sorenson & Harrell, 2021). When culture is not incorporated, clients can report feeling frustrated and misunderstood, risking premature dropout and resistance to future help-seeking (Marsiglia & Booth, 2015; Rathod et al., 2018). Evidence indicates that culturally adapted interventions are more effective for culturally diverse clients (Cabassa & Baumann, 2013; Rathod et al., 2018). Notwithstanding, many interventions have been developed and tested for WEIRD (Western, educated, industrialised, rich, democratic) populations (Rad et al., 2018) and although not necessarily suitable, many interventions are used with non-Western clients (Bernal et al., 2009; Cabassa & Baumann, 2013). As such, it is important to explore the cultural suitability of Western intervention models with different cultural populations to better meet the needs of diverse clients (Cabassa & Baumann, 2013).

Schema therapy (ST) is a Western-developed approach gaining worldwide acceptance (International Society of Schema Therapy [ISST], 2019) which was developed for complex and chronic issues (Young et al., 2003). ST emphasises the childhood origins of distress and the use of experiential techniques (i.e., Imagery Rescripting and Chair Work). ST also incorporates a unique therapy relationship with Limited Reparenting. In Limited Reparenting, the therapist adopts the role of a “good” parent, defined as a role model who provides corrective emotional experiences by meeting core emotional needs that were not adequately met in the client’s life, to promote the development of the Healthy Adult Mode (HAM; Young et al., 2003). One key component of ST is Early Maladaptive Schemas (EMS; or Schemas); broad cognitive patterns about the self and interpersonal relationships that are elaborated across the lifespan and distressing when triggered (Young et al., 2003). When an EMS is triggered, an individual’s coping mechanism results in the activation of a Mode; a transient coping response that can be adaptive or maladaptive (Young et al., 2003). The goal of ST is to reduce EMS activation and strengthen the Healthy Adult Mode (HAM). The HAM is an emotionally regulated state with appropriate adult functioning (Young et al., 2003). A growing body of the literature supports the effectiveness of ST in reducing EMS and symptoms across a range of mental health disorders including personality disorders (Zhang et al., 2023), eating disorders (Joshua et al., 2023), anxiety (Peeters et al., 2021), and depressive disorders (Körük & Özabacı, 2018).

Some evidence supports the cross-cultural application of schema theory; particularly, the Young Schema Questionnaire (YSQ; Young, 2005) has been validated in various countries (e.g., Oettingen et al., 2018; Sakulsriprasert et al., 2016). Support for the universality of positive Schemas has also been established in the development and validation of the Young Positive Schema Questionnaire that used samples from both Eastern and Western populations (Louis et al., 2018). However, the evidence base for ST in non-WEIRD contexts is scarce (Arntz et al., 2021). In a recent exploratory qualitative study, Mao et al. (2022) investigated perceptions of the suitability of ST amongst Asian therapists. They found that core ST concepts were acceptable but required substantial cultural adaptations. Lian et al. (2024) reported the effectiveness of culturally adapted ST for addressing trauma in a Malaysian context. Their adaptations involved developing a continuum of techniques to address EMS and Modes, beginning with “emotionally gentle” approaches (i.e., art therapy and dream-work) to more “emotionally overwhelming” techniques (i.e., Imagery Rescripting and Chair Work). Two recent case studies have reported the benefits of culturally adapted ST for Middle-Eastern clients (Barbieri et al., 2022; Henry & Nasreldin, 2024). Cultural adaptations included language translation, consideration of cultural values and contextual stress, and understanding the client’s EMS in their cultural and religious contexts. Although case studies provide useful clinical insights, they do not allow evaluation of intervention effects, warranting further research.

Despite the importance of cultural responsiveness, there is minimal guidance for clinicians on achieving this in ST. A major challenge for clinicians in delivering evidence-based interventions (EBIs) is the tension between using a standardised protocol versus adaptation for clients’ cultural needs (Gallardo et al., 2009). Models for cultural adaptation of EBIs have been developed to address this concern. Sorenson and Harrell’s (2021) four-domain cultural adaptation model (CAM4) has particular utility, providing guidance on potential modifications rather than a “one-size-fits-all” approach (Table 1).

**Table 1.** 4-domain cultural adaptation model (CAM4; Sorenson & Harrell, 2021.).

Domain	Domain description
Development and equivalence processes	Adaptations maintain treatment fidelity alongside cultural appropriateness. “Bottom-up” approach, consulting community stakeholders and population members. Potential adaptations follow intervention design processes involving piloting, re-examination, refinement, and final effectiveness test.
Cultural context and content	Meaningful incorporation of cultural values, beliefs, and understandings of wellness/illness. Consideration of local community, socio-political, and linguistic context. Surface adaptations (e.g., mental health psychoeducation to mitigate stigma) versus deeper adaptations (e.g., incorporation of cultural ideas, values, beliefs).
Engagement efforts	Addresses factors affecting intervention engagement including communication styles, role expectations, and introducing the intervention in communities where there is mistrust, stigma, and/or unfamiliarity.
Cultural competence	Clinicians’ cultural awareness, attitudes, knowledge, skills, and cultural humility. Monitoring implicit bias, attitudes and self-reflection, and personal characteristics. Clinician credibility as perceived by the client and/or community. Agency/system cultural competence involving ethical considerations and re-evaluation of traditional practice expectations.

## Cultural considerations for schema therapy

While there are no documented cultural adaptation guidelines for ST, the CAM4 points to aspects of ST that warrant exploration, as illustrated in Table 2.

**Table 2.** Cultural considerations for schema therapy: context and content.

Aspect of schema therapy	Cultural literature	Potential cultural consideration(s) for schema therapy
Grounded in Western values (independence, autonomy, boundary setting; Young et al., 2003).	Hofstede’s (2011) cultural dimensions describe patterns of behaviour that may vary across cultures, (e.g., collectivistic versus individualistic and small versus large power distance).	Different cultural values and behavioural norms. Various expectations of Healthy Adult Mode.
Experiential techniques meet core emotional needs (Young et al., 2003). Assumes that expressing and experiencing emotions is adaptive and emotional suppression is maladaptive.	The collectivistic nature of Chinese culture and associated desire to save “face” made expressing emotions in ST difficult for some Asian clients (Mao et al., 2022)	Emotion display rules vary across cultures. Some clients may struggle to engage in experiential techniques.
Clients must express anger at the imagined parent during experiential techniques to meet core emotional needs (Young et al., 2003).	Filial piety made it difficult for Chinese clients to express anger towards and confront the imagined parents (Mao et al., 2022).	Confronting parents’ behaviours may be perceived as disrespectful for some clients. Some clients may experience guilt for disrespecting parents.

### Cultural competence

Clinicians have an ethical duty of care to be culturally responsive (Ridley et al., 2021). To achieve this, clinicians can monitor their implicit biases and maintain awareness of the cultural and personal differences between themselves and their clients (Sorenson & Harrell, 2021). Supervision is an effective forum for monitoring bias, suggesting that appropriate supervision could be important for ensuring cultural competence in ST (Edge & Lemetyinen, 2019; Soheilian et al., 2014). Similarly, professional development can help clinicians learn about cultural adaptations of ST. As yet, no research exploring ST clinicians’ perspectives of their own cultural competence, training or support needs (such as supervision) has been conducted.

## Current study

A useful starting point in examining the cultural suitability of an intervention is to engage with practising clinicians (Sorenson & Harrell, 2021). Qualitative interviews are suitable for investigating lived experiences of clinicians (e.g., Bengtsson et al., 2015) and have been used to explore the cultural acceptability of ST (e.g., Mao et al., 2022). Thus, the current study aimed to (a) understand clinicians’ perspectives about the cultural suitability of ST; and (b) explore whether cultural adaptations to ST are necessary, and if yes, what adaptations? A heterogeneous sample of clinicians from diverse geographical locations was considered appropriate. Findings will illuminate potential considerations for improving the cultural responsiveness of ST and inform future research.

## Method

### Design

A qualitative exploratory design with semi-structured interviews and reflexive thematic analysis (RTA; Braun & Clarke, 2022) was used. An interpretivist paradigm was adopted for this research which posits that reality is subjective and that multiple realities exist, each shaped by the cultural, social, and historical contexts within which they occur (Crotty, 1998). Ethics approval was obtained through the Monash University Human Research Ethics Committee (ID: 28477) before commencing recruitment.

### Participants

Members of the ISST were invited via email to participate. Participants must have either completed or been undertaking ST certification. Eighteen clinicians completed an online expression of interest survey via Qualtrics and were emailed to arrange an interview. Six did not respond and one cancelled, leaving a final sample of 11 practising ST clinicians from nine countries. Due to time constraints for this project, no further participants were recruited. Mean age was 42.4 years (*SD* = 10.8, range = 25–62) with mean 6.1 years of ST practice (*SD =* 4.0, range = 2–15). Participants’ cultural identity was confirmed verbally during interviews. Table 3 shows participant demographics.

**Table 3.** Participant demographics.

Pseudonym	Gender	Age	Location of practice	Ethnicity	Certification status	Years of schema therapy practice
Sarah	Female	31	Australia	Vietnamese	Undertaking standard	5
Isabel	Female	62	United States	Latina	Undertaking standard	7
Hanna	Female	43	Poland	Polish	Advanced	10
Alana	Female	39	Australia	Maltese	Undertaking standard	6
Elisah	Female	57	Israel	Israeli	Advanced	15
Aarav	Male	38	Southeast Asia*	Southeast Asian*	Standard	2
Amalia	Female	43	Netherlands	Turkish	Advanced	9
Edwin	Male	37	Northeast Africa*	Northeast African*	Advanced	4
Evelyn	Female	40	Romania	Romanian	Advanced	5
Oliver	Male	25	Brazil	White Brazilian	Advanced	2
Daniel	Male	51	United States	European American	Standard	2

* Region instead of country to maintain participant confidentiality.

### Data collection

Interviews were conducted by the first author, IM, via Zoom. Average duration was 61 minutes (range = 48–85 minutes). The interview questions were informed by the research questions and guided by past research that has explored the cultural suitability of EBIs (e.g., Naeem et al., 2019, Rathod et al., 2019). To ensure the relevance of the questions, draft interview schedules were reviewed by two practising Schema Therapists, resulting in minor wording changes and the addition of one question. The finalised interview guide is provided in the Appendix. Interview questions explored participants’ perspectives on the suitability of ST (e.g., “In your experience, has the ST model been applicable to your clients?”), and participants’ cultural background (e.g., “How, if at all, has your own cultural context influenced how you practise ST?”). Probes helped obtain more detail (e.g., “Can you tell me more?”). Interviews were recorded with participant consent and transcribed using Otter.ai. The first author corrected errors and removed potentially identifying details. Transcripts were emailed to participants to review and amend if they wished. Two participants made minor clarifications (e.g., for some South-East Asian clients, the Angry Child Mode is also considered inappropriate to express beyond sessions in their “collective communities”).

### Analysis

Analysis was guided by the six phases of RTA; a qualitative method designed to identify, analyse, and report themes within data (Braun & Clarke, 2022). Familiarisation with the dataset involved listening to interview recordings and reading accompanying transcripts multiple times, while making brief notes about initial reactions and basic analytic ideas (e.g., a brief note from Amalia’s transcript: “Some clients struggle with accepting affection during Limited Reparenting because it feels condescending and unrealistic”) to strengthen engagement with the data.

Once familiarity was established, key ideas in each section of the transcripts were coded using an inductive, bottom-up approach in which there was no attempt to fit the data into an existing theory (Braun & Clarke, 2022). Initially, semantic codes were produced, but as familiarisation and immersion with the data evolved, latent codes were produced (e.g., “difficulty accepting affection” was refined to a latent code of “cultural emotion display rules discourage overt displays of affection”). Once all the interviews were coded, codes were collated and relevant interview excerpts were compiled for each code.

The following three phases focused on theme development. First, initial themes were created by grouping together potentially related codes, which were then reviewed with the research questions in mind. This phase was repeated, which allowed for movement between transcripts, codes, and themes to maintain closeness with the data and promote deeper understanding of participant accounts. This iterative process was adopted until themes were refined and clearly demarcated. Developed themes were understood using the CAM4 (Sorenson & Harrell, 2021) and then composed into narratives with illustrative participant quotes. When a theme is discussed, some quantifying language will be used to discuss its prevalence across the sample to provide an indication of its’ strength or consistency. The term “most” refers to occurrences of themes within nine to 11 participants’ accounts, “many” refers to six to eight participants, “some” reflects three to five participants, and “few” denotes up to two participants.

### Rigour

Trustworthiness was ensured as follows: Interview transcripts were analysed and coded multiple times by the first author, IM, to ensure prolonged iterative engagement. Transcripts were also double-coded to enhance understanding and interpretation by either the third author or an independent researcher who was a PhD candidate conducting ST research. Participants were emailed a summary of preliminary themes as a quality check; all participants reported satisfaction with the summary. These strategies combined helped enhance credibility. Regarding transferability and confirmability, due to the centrality of the researcher in RTA (Braun & Clarke, 2022), the authors’ positionalities inevitably informed the analytic process. Further, the interpretivist paradigm acknowledges that interpretations of data are a co-construction of meaning with participants (Crotty, 1998). Consequently, another researcher conducting RTA with the same dataset may produce a different analysis. Nonetheless, rich descriptions of the research context, participants, and findings have been provided, allowing readers to determine the transferability of the current findings. The six phases of RTA (Braun & Clarke, 2022) were followed to establish dependability. Finally, reflexive awareness was maintained to further enhance trustworthiness.

The first author is a second-generation Indian Australian, completing her PhD and working as a provisional psychologist. She brought a perspective shaped by her cultural background and experiences of attending therapy and psychological training that did not always align with her cultural values. Her lack of professional affiliation with ST facilitated an objective exploration of its cultural suitability. Nonetheless, her identity as a person-of-colour heightened her sensitivity to cultural discrepancies in ST. This duality allowed her to approach the data with both insider and outsider perspectives. The second author is a registered counselling psychologist experienced in qualitative research, private practice, and postgraduate counsellor and psychologist training and supervision. The third author is a registered psychologist and ST practitioner with qualitative research experience. The fourth author is a senior research fellow and provisional psychologist with cross-cultural research experience. The first author completed all the interviews and completed most of the data analysis in collaboration with the other authors.

The first author, IM, maintained a reflexive journal to record initial reactions post-interviews and reflections during data analysis. The journal helped explore how her positionality influenced the research process. IM initially expected that all participants would share experiences of cultural incongruence in ST; however, some participants reported ST was culturally suitable, contradicting data expectations. This led IM to understand the need for a contextualised view of participants’ experiences, for example, participants who reported ST as culturally suitable worked with clients of similar cultural backgrounds. IM’s positionality also influenced the initial coding stage, as she focused heavily on participant accounts that mirrored her experiences, particularly relating to clinicians-of-colour like Isabel and Sarah. Upon reflection and supervision, this pattern was addressed, allowing for a broader exploration of perspectives through further rounds of coding. Themes were regularly discussed in supervision, helping authors to further separate their preconceptions from the analytic process.

## Results

Overall, participants endorsed the benefits of ST. However, views on the cultural suitability of ST were mixed. Some said that ST was “individualistic” and most said that ST required cultural adaptations. Interestingly, Evelyn explained that she only started recognising a need for cultural adaptation in ST once she started working with clients from a different cultural background to her own: “you are becoming more aware of the differences in culture when you’re working with different cultures”. Some participants commented that ST had a “large variety of tools” (Amalia), which helped select and adapt techniques for diverse clients. In contrast, a White Brazilian male therapist (Oliver), working mainly with White Brazilian clients, said “the ST model is really comprehensive and it fits all of these differences in culture within the model”, indicating that he saw no need for cultural adaptation.

Two main themes were identified, each with subthemes: (i) Cultural Context and Content and (ii) Cultural Competence. Themes are presented below with representative participant excerpts.

### Cultural context and content

A number of themes were identified that highlighted ways in which ST may not be culturally suitable for all clients: (i) Incongruence with Cultural Norms of Emotional Expression, (ii) “Maladaptive” or “Adaptive”, But in What Context?, and (iii) Issues Related to Familism when Addressing Parent/Critic Modes.

#### Incongruence with cultural norms of emotional expression

Many participants discussed an incongruence between ST and some cultural norms of emotional expression. Sarah observed that some clients with Asian backgrounds were “taught growing up that you don’t dwell on the past, you got to be strong, you got to move forward … [which] clash[es] with the whole idea of ST” and its focus on core emotional needs. Similarly, Isabel reflected that for some South American men, “emotions are seen as a weakness” so emotions are expressed through actions rather than words, an observation echoed by Aarav in most of his South-East Asian clients. Participants explained that clients’ difficulty expressing emotions created challenges for experiential interventions, especially chair work:

I think it’s easier with the imagery [rescripting] because … I think it’s like, “saving the face” as the Japanese are saying. Because, you know, if you are in imagery, you have your eyes closed and the other person doesn’t see what’s happening but when you are on chairs, you feel more exposed. So, the shame feeling gets more triggered in the chair work than in the imagery exercise.

(Evelyn)

Alana described a similar observation:

… chair work I find a little challenging … because you’re asking [clients] to act something out in a room and they feel like it’s a bit dramatic and different and can be a little confronting … They really struggle with that.

Few participants reflected that despite clients’ initial difficulties it was “very liberating” (Evelyn) when they eventually learnt to express emotions. Sarah expanded on this:

I would definitely say that clients from an Asian background, who tend to be quite emotionally deprived, find that ST works well for them; because they go through their own journey of actually learning how to identify and express their feelings, and knowing that it’s okay, because it’s really scary doing that. And it’s scary to think about putting down the walls and being vulnerable.

Sarah added that when her clients viewed her as competent, it contributed to a stronger therapeutic relationship which ultimately helped her clients feel more comfortable expressing emotions: “…as long as there’s that trust [in the relationship] and you can hold a safe space and demonstrate that you can have the strength to be able to be present with whatever they bring”.

Strategies for assisting clients with emotional expression were considered important and discussed by most participants. Some participants used creative techniques, for example: “when you draw, you connect to that Child Mode” (Hanna). Elisah incorporated dream work into Imagery Rescripting for some clients:

… we started doing this imagery work with [client’s] dreams, using her dreams as images … and through the images of the dreams, we could be there together and change the way she was reacting in her dreams, like I would do in regular Imagery. [Rescripting]

Few participants reported asking clients to empathise with other people’s experience because “when we think back to the collectivist societies, people … love the way that groups come to support them” (Aarav). Few participants observed that focusing on physical symptoms can benefit clients who tend to “believe in somatic disorders, not psychological [disorders]” (Edwin). Few participants discussed the benefits of metaphors for making conversations about emotions feel less stigmatising: “I use a lot of metaphors to teach about [Schemas and Modes]” (Amalia). In contrast, Oliver explained that experiential techniques “fit really well with Brazilian culture” due to its expressive nature.

#### “Maladaptive” or “adaptive”, but in what context?

Most participants reflected on the terms “maladaptive” and “adaptive” as used within ST. Specifically, participants explained that there were differences in the idea of the HAM across cultures. For example, Hanna reflected:

… the idea of the Healthy Adult [Mode] will be a bit different in different countries … [consider] power distance; Is the society [in a] need to be kind of equal or is it more like a need to maintain hierarchy? And, how do you find yourself in it? So, that would, I guess, shape the Healthy Adult. [Mode]

Participants also explained that Modes “are more or less pronounced in different cultures” (Hanna). This was clearly articulated by Sarah:

Compliance-Surrenderer [Mode; CSM] is highly encouraged in a lot of cultures because to be seen as selfless and humble … [is] really encouraged … And if you look after yourself, something that’s seen as being individualistic, you’re straying from the family unit. Everything’s around being a unit and looking out for each other and when some clients want to kind of break off and do their own thing … it’s seen as going against the grain.

Similarly, few participants described how some clients related to the Detached Protector Mode (DPM). Sarah explained:

[With the DPM], you kind of numb your feelings, you go on autopilot, you go through the motions of each day, but you’re not really present … culturally, some of these clients see that as being strong … to face adversity and get through and not think about it and not deal with it.

As such, Isabel reflected that “[clients] see it as a weakness if you take [the DPM] away”.

Some participants noted the incongruence between the standard description of the HAM and some cultural norms, which left some clients feeling “defective” (Alana) or that “their thoughts are wrong” (Aarav). Edwin elaborated: “ST is making [clients] nervous … making them afraid because it’s challenging their beliefs, challenging their traditions … it makes them feel terrible … They think ST is destroying what they believe and they will not accept this”.

Many participants agreed that it was important to understand the HAM within the clients’ cultural context, as illustrated by Isabel:

Many Latina women are self-sacrificing. That’s perceived as respectful and as a core value … So, where is self-sacrificing not working within the cultural context of that person that I’m seeing? And [I try] not to put a worldview, an American worldview, of independence and non-self-sacrifice.

#### Issues related to familism when addressing Parent/Critic modes

Participants described challenges when implementing ST techniques with clients who valued familism. Few participants revealed that even after instances of childhood abuse, clients from some cultures still believed that ”… you have to obey [parents] … [and] respect [parents] all the time” (Edwin). These deep-rooted values of familism were evidenced by participants’ accounts of their clients’ beliefs that “they’d be punished” (Elisah) for confronting their parents’ behaviours during experiential techniques. Further, some participants noted that some clients experienced guilt when encouraged to confront their inner Critic Modes: “getting rid of the parental voice, it’s like you’re betraying your family” (Hanna). Edwin explained that many of his clients felt that perceiving the therapist as a caregiver figure was akin to “betraying” their parents:

… when we found that Limited Reparenting and Chair Work and Imagery Rescripting is going well [for] him, and he is feeling like I am a caregiver for him, he is talking punitive to himself, or blaming himself because he let himself get closer to me. Because it’s not right. It’s not allowed.

(Edwin)

Some participants described some clients’ dilemmas when personal needs clashed with a sense of duty to meet parent expectations:

Clients where their families have worked their asses off, essentially, to give the opportunity, I find then that expectation that “you owe this to us” is quite strongly embedded. So, there’s an extra element of guilt and self-sacrifice … That’s where those Self-Sacrificing and Subjugating Schemas are quite strong and they feel like they need to be quite compliant with the family and cultural expectations.

(Alana)

Highlighting the value of family connection, some participants reflected that for some clients, especially those from South-East Asian backgrounds, Imagery Rescripting resulted in “the child in the image bond[ing] with the parent, rather than feeling protected” (Alana) by the therapist. Conversely, therapists from Romanian and White Brazilian backgrounds (Evelyn and Oliver respectively), who primarily worked with clients from their own cultures, reflected that clients often responded positively when the therapist acted as a protector.

Participants discussed strategies to improve clients’ experience when working with Parent/Critic Modes. Some participants emphasised the importance of a compassionate approach when addressing Parent/Critic Modes. For example, Elisah described her “standard” Imagery Rescripting: “I will be really [fierce] … I will stand there by the child, and I will stop the parent. I will not let the parent in the imagery exercise harm this child again”. However, when working with clients who valued familism, she reflected “… you cannot do that, you know? He’s the father, she’s the mother. You cannot stand up to her and talk to her like that”. Some participants reported that they explain to clients: “we’re not rejecting the Parent Mode, we’re just softening it [and] changing it to a more loving, caring voice” (Hanna). Similarly, some participants inform their clients that ST provides an opportunity for emotional needs to be met: “we’re not sitting here for you to criticise your family … you’re just describing what you felt as a child” (Elisah). Few participants also recommended replacing the term “Parent Mode” with “Critic Mode” to make it feel less disrespectful when exploring such Modes.

Some participants commented on the broader value of age hierarchies. For example, Sarah (aged 31) said “culturally, for someone younger than their parents and grandparents to enter the image [in Imagery Rescripting], it’s breaking the pecking order. And that’s not realistic to [clients]”. To address this age hierarchy, participants described using alternative meaningful adult figures, stating that “there’s usually at least one healthy adult in [the client’s] life” (Sarah) who can enter the imagery instead of the therapist. Participants added that grandparents often make good substitutes because they are “someone that their parents would listen to” (Sarah). Notably, not all therapists felt it was important to address age hierarchies, for example, Oliver (White Brazilian, aged 25) said his age had not impacted his clients’ comfort with techniques.

### Cultural competence

The second theme related to clinicians’ Cultural Competence in ST: (i) Perceptions of Reduced Confidence and Competence in Providing Culturally Responsive Practice, and (ii) Clinicians’ Cultural Values Impacting ST Delivery.

#### Perceptions of reduced confidence and competence in providing culturally responsive practice

Many participants reflected on their perceived self-efficacy with culturally responsive ST. Oliver, whose “clients are all white”, felt confident implementing standard ST with all of his clients, saying the “schema model fits perfectly” with his Brazilian culture. In contrast, some clinicians reported concerns about adapting ST, partly due to a fear of compromising treatment fidelity. Edwin (four years practice) said he was “trying to [use] the techniques as they are designed”. Similarly, Isabel (seven years practice) said: “I feel like I’m so new to ST, that, ‘should I be adapting anything to the client?’” Alana (six years practice) who often incorporated cultural adaptations wondered whether she was “doing ST wrong by being so flexible”. Amalia, a ST supervisor who assesses videos submitted for accreditation, reflected that clinicians display “anxieties in the beginning of their profession … and they do not exit from their plans of the sessions a lot”. Tension between adhering to standardised ST and making necessary cultural adaptations was evidenced by Isabel’s experience with a “supervisor [who] was not impressed” with her adaptations, further exacerbating her self-doubt and anxiety around culturally responsive practice. This tension was also present in a few participants' reflections on when therapy did not go to plan:

He dropped out of therapy. He didn’t like what ST had to say about it because he didn’t want to change his views … and I don’t know whether that’s because I maybe didn’t approach it in the right way.

(Sarah)

Few therapists reported that developing cultural adaptations was an iterative learning process, requiring more investment than the standardised application of ST:

The thing I must say [is] that [ST is] really hard … It use[s] a lot of trial and errors … and you know, it cost me a lot of time and a lot of effort more than [other] people … It uses a lot of effort.

(Aarav)

Most participants discussed the need for better support in making cultural adaptations. Specifically, many participants indicated a need for culturally informed ST training, stating that there was “no reference to cultural factors in the [ST training] material” (Daniel). However, participants acknowledged that culturally diverse professionals attended ST training workshops, facilitating diverse perspectives on the cross-cultural application of ST. Some participants reflected that their experiences of ST supervision were not always culturally responsive, and many participants highlighted that ST resources often used Western examples that were not always relatable for clients.

#### Clinicians’ cultural values impacting schema therapy delivery

Participants reflected on the impact of individual cultural values on their ST delivery. This included cultural norms of familism and their own expectations of the HAM.

Few participants’ own values of familism contributed to a conflict between cultural respect and therapeutic efficacy:

My Latina therapist [part] is going to be very respectful to [parents] … My American therapist part is going to be like, “You’re letting culture affect your ability to do Imagery Rescripting. You’re being too sensitive, you’re being too cautious. You [have] got to get over it”.

(Isabel)

Some participants acknowledged the importance of setting aside personal expectations of the HAM and adopting a person-centred approach when working with clients who held different expectations of the HAM. For Alana, this involved exploring and understanding clients’ values, rather than imposing change:

I find I get triggered a little bit by my values around … being independent and self-sufficient … And there’s some cultures where … it’s almost [an] obligation that you’re there to look after the family and that’s your role and that you don’t have any independent sense of self … [That] is probably the cultural belief systems and values I struggle the most with … And I have to be very conscious to not come in as a protector, to try to save that person or to challenge it too directly … a lot of it is holding my value system and not voicing it and going back to the client and thinking, “what’s the impact on them?”… [I’m] not trying to impose my value systems on them, because they’ve got a right to those, even if I think it’s doing them harm.

Evelyn described a similar person-centred approach:

I would try, as much as I can, to remain in my Healthy Adult [Mode]. And to be curious around [my client’s] values. And to invite the client in a Healthy Adult [Mode] with me, not to question but more to explore those values … Respectfully inviting them into a healthy adult perspective and asking them, “Look, I don’t want to contradict this value of yours. I know it’s important for you, but I just want to look at it from the perspective of the problem you came in therapy with”.

Similarly, Daniel reflected on a client’s values that clashed with his own: “I personally react to that as though I’m dealing with a pathology … So, I’ve had to learn how to maintain a supportive bond with [the client] and separate it from my values”.

## Discussion

This study was an exploratory study of 11 clinicians’ perspectives on the cultural suitability of ST. Interviews revealed that while the ST model is generally acceptable, adaptations may be required. The current findings should be interpreted within the context of the participants, all of whom were completing or had completed ST accreditation. This context meant that participants had undergone extensive training in ST and were invested in its practice. As such, their perspectives were valuable in understanding the application and suitability of ST; however, this context may have also influenced their responses. Themes are now discussed in relation to the literature and the CAM4 (Sorenson & Harrell, 2021).

### Considerations for cultural context and content

One key finding in the present study was a clash between some cultural emotion display rules and core ST principles. Some participants identified that clients whose cultures emphasised resilience over dwelling on past experiences could struggle with the focus on core emotional needs, making it difficult to engage in experiential techniques. This finding aligns with Mao et al. (2022) findings and broader collectivist values, where suppression of powerful emotions, particularly anger, is desirable to maintain group harmony (He et al., 2021; Hofstede, 2011). Further, the present findings suggest that in some cultures, it is preferable to show affection through helpful actions and advice, rather than verbally or physically. This finding is unsurprising given that emotion display rules influence how affection and care are communicated (Wang & Lau, 2015). While difficulties with emotional expression were primarily explored in the current study regarding non-WEIRD cultures, research suggests that discomfort may be experienced by clients from WEIRD cultures too, although the context in which this is elicited may vary (Fonseca et al., 2023). For example, Fonseca et al. (2023) findings showed that British participants (WEIRD) strongly endorsed beliefs about the unacceptability of emotional expression while Brazilian participants (non-WEIRD) endorsed the need to control their emotions, especially negative emotions. This underscores the importance of clinicians encouraging clients to explore and express emotions while exploring personal attitudes towards emotions. Our findings suggest that when clients feel supported and understood, they can benefit greatly from ST. That is, prioritising a strong, trust-based therapeutic relationship, especially when working with clients who may resist emotional vulnerability, can enhance clients’ comfort with emotional exploration and expression (Gülüm & Soygüt, 2022).

Participants in this study discussed strategies used to navigate barriers to emotion-focused work. Creative methods (e.g., drawings and dream-work) can facilitate non-verbal expression of Modes and emotional states (Heath & Startup, 2020; Lian et al., 2024). Perspective-taking utilises the strengths of collectivist cultures and has previously been found to be more effective in collectivist cultures than individualist groups (Liddell & Williams, 2019). Additionally, focusing on physical symptoms as a pathway to emotional exploration may be particularly beneficial for clients who are more comfortable discussing somatic symptoms (Heath & Startup, 2020).

While there were some suggestions that the emotional-focus of ST aligns well with Brazilian culture, it was also suggested that South-American men may view emotions as a weakness, highlighting intracultural variability in emotion display rules (McSweeney, 2002). This suggests that while some clients may find it difficult to engage in experiential techniques, others may feel comfortable, emphasising the importance of individualising ST for the client.

Another major finding in the present study was the influence of cultural norms on the perceived adaptiveness of Schemas and Modes; a finding replicated from Mao et al. (2022). Current participants reported an incongruence between the standard HAM and certain cultural norms. Hofstede (2011) argued that differences in behavioural norms and expectations may impact notions of cultural “appropriateness” and “adaptiveness”. As such, some clients may experience distress when ST challenges their deeply held cultural beliefs, increasing the risk of disengagement (Rathod et al., 2018). For example, the HAM is expected to fight and replace maladaptive coping Modes such as the DPM and CSM (Young et al., 2003). However, the present findings suggest that the DPM is often considered a strength in some cultures, demonstrating resilience (Hofstede, 2011). Similarly, the CSM was reported as appropriate in some cultures by participants in the current study, aligning with broader collectivist perceptions that self-care and autonomy indicate straying from the family unit (Hofstede, 2011). Consequently, considering these Modes as “maladaptive” and attempting to reduce or control the DPM or CSM may be met with resistance (Mao et al., 2022). These findings underscore the importance of exploring the influence of broader social and cultural norms on Schemas and Modes. For example, in high power distance cultures, characterised by a hierarchical structure of respect (Hofstede, 2011), the development and adaptiveness of the CSM may be affected. As such, descriptions of “maladaptive” and “adaptive” need to be examined with a cultural lens as our findings suggest that Schemas and Modes can intersect with cultural values, influencing their development and perceived adaptiveness. In line with Hays’ (2009) recommendation for Cognitive Behaviour Therapy, ST clinicians are well advised not to challenge core cultural beliefs as clients may perceive this as disrespectful or naive. Instead, the function of Schemas and Modes can be explored, including their advantages and disadvantages, within the client’s cultural context using a dimensional approach to adaptiveness.

Participants in the current study also discussed cultural considerations for working with Parent/Critic Modes. Although Young et al. (2003) argued that clients must express their anger towards parents during experiential techniques, this premise is misaligned with expectations of parent–child relationships in collectivist cultures (Hofstede, 2011). Present findings suggest that some clients feel compelled to obey and respect their parents, regardless of parents’ actions, highlighting a clash between cultural expectations and therapeutic goals in ST. Similar to Mao et al. (2022), participants in this study indicated that the guilt-inducing Critic is activated more frequently in collectivist cultures when confronting Parent/Critic Modes, suggesting that for some clients, the Parent/Critic Mode is not just an internalised critic but a revered figure whose authority must not be challenged. Current findings also highlighted some clients’ dilemmas between maintaining parental reverence versus meeting their own emotional needs. This aligns with Dinh and Kalaja (2023) who found that while Asian-American young adults criticised traditional cultural norms, they nevertheless complied with parental expectations to maintain parental acceptance. As such, challenging Parent/Critic Modes may not always be acceptable.

Present findings indicate a need to develop ways of working with Parent/Critic Modes that are tailored to individual clients’ level of comfort. Some clients may prefer clinicians adopting a respectful tone when addressing the imagined parent, such as acknowledging that while parents may have had good intentions, the clients’ needs were not always met (Mao et al., 2022). Explaining the purpose of experiential techniques may help reduce clients’ feelings of guilt (Josek et al., 2023); for example, explaining that the purpose is to provide corrective emotional experiences, not to vilify or replace parents. The use of an alternative meaningful adult figure (e.g., grandparents) in imagery exercises is another culturally responsive strategy that leverages the importance of age hierarchies to facilitate therapeutic progress (Huisy-Howland, 2021; Mao et al., 2022).

### Clinician cultural competence

Participants offered valuable insights into their perceived self-efficacy in providing culturally responsive ST. While one participant felt confident in the standardised ST framework, most shared the belief that ST was a Westernised approach requiring cultural adaptation. However, participants expressed concerns about adapting ST, due partly to a fear of not maintaining intervention fidelity, a common experience among clinicians (Gallardo et al., 2009). This apprehension seemed to be compounded by participants’ self-doubt in their cultural competence, particularly for early-career clinicians. Clinicians who are newer to ST may be more vulnerable to criticism which could prevent them from making the necessary adaptations (Young et al., 2003). These findings warrant attention as lower clinician confidence predicts poorer client outcomes (Soheilian et al., 2014), more strongly impacting the effectiveness of ST for diverse clients.

A need for culturally informed ST training and supervision was also raised by participants. Participants’ reports that ST training materials do not adequately address cultural factors, their experiences with supervision that were not culturally responsive, and the use of Western examples in ST resources, reflects a gap in current professional development opportunities. While the presence of culturally diverse professionals in training workshops provides some exposure to cross-cultural perspectives, this was described as insufficient. Past research indicates that supervisors who receive supervision from multiculturally competent supervisors feel more confident modifying approaches, recognising personal limitations and understanding their culturally diverse clients (Soheilian et al., 2014). This highlights the need for more culturally inclusive and representative training and supervision practices in ST.

These findings have several implications. Firstly, ST training programmes may benefit from integrating cultural competence modules that address potential considerations for diverse clients. This could also include supervisor training to promote culturally responsive supervision. Further, developing culturally appropriate ST resources, including case examples reflecting a broad range of cultural experiences, can equip clinicians with tools to better engage diverse clients (Benuto et al., 2021).

Participants in this study also reflected on the impact of their own cultural values on their ST practice. One key point raised was the internal conflict clinicians’ experienced when their own values clashed with the ST model. Isabel’s reflection on her dual identities – Latina and American – highlighted this conflict. Her Latina identity emphasised parental reverence which can hinder her from implementing techniques in a standardised format, while her American identity encouraged her to overcome cultural considerations and adhere to the standardised format. This highlights the challenge in balancing cultural respect without compromising ST effectiveness.

Another critical point discussed by participants was the importance of reflecting on personal views of the HAM. Alana’s difficulty with her values around independence versus family obligation illustrated the challenge of managing countertransference. Her need to avoid imposing her values on her clients, even when she deemed her clients’ values as harmful, emphasises the delicate balance clinicians must maintain between respecting clients’ cultural beliefs and promoting their wellbeing. Instances of countertransference are common among schema therapists (Pilkington et al., 2022). A recent study found that schema therapists’ Schemas and Modes were often activated when clients’ childhood experiences reminded them of their own, resulting in some clinicians becoming argumentative, disengaged or detached, or overstepping their responsibilities and adopting a “rescuer-role” (Pilkington et al., 2022). This, combined with present findings, underscores the importance of therapists to continuously reflect on their practices and manage personal values. ST clinicians may find it beneficial to engage in self-reflection, refocus on the client’s story, and engage in supervision, training, and personal therapy (Benuto et al., 2021; Pilkington et al., 2022).

### Limitations and suggestions for future research

This study has some limitations to consider. First, clinicians’ perspectives on the cultural suitability of ST may not reflect clients’ experiences. Further research evaluating clients’ experiences of ST would complement the present findings. The small sample size was appropriate for this exploratory study, but studies with larger sample sizes using other methods (e.g., survey) could enhance our understanding. Future studies with homogenous samples would help understand the suitability of ST in specific cultural groups (e.g., Mao et al., 2022). Participation was voluntary, creating potential sampling bias for clinicians willing to share their views (e.g., a vested interest in the topic). Nonetheless, the sample had diversity including country of practice, clinician ethnicity, and years of ST practice. Further, the sample was limited to a specific subset of clinicians who were completing or had completed ST accreditation to ensure participants had comprehensive knowledge of the therapeutic process. However, these inclusion criteria meant that the perspectives of clinicians who may have found ST culturally incompatible and thus did not pursue accreditation were not captured. These voices are critical to understanding the full range of perspectives on the cultural suitability of ST and future research is warranted with such a sample. Another limitation was that for some participants, English was not their first language, creating some difficulty articulating their reflections despite being enthusiastic about the interviews. While participants were asked clarifying questions and provided opportunities to amend their transcripts, future studies may benefit from a translator to ensure concepts are articulated clearly. Finally, similar to Mao et al. (2022), potential cultural adaptations to ST identified in the current exploratory study must be empirically evaluated.

## Conclusion

Cultural responsiveness in therapy is an ethical imperative. Clinicians in this study provided their perspectives on the cultural suitability of ST as experienced in their professional practice. A culturally adapted intervention framework is yet to be developed for ST and previous research has scarcely investigated the cultural suitability of ST. Current findings indicate that despite the flexibility of the ST model in allowing ad-hoc cultural adaptations, more systematic research is needed to establish evidence-based cultural adaptations. In the interim, ST clinicians are well advised to adopt a person-centred stance in their practice, in which the cultural context of each client is actively explored and incorporated into the therapeutic process. This approach ensures that the interventions used in ST are culturally suitable and responsive to the needs of individual clients, ultimately improving its effectiveness. Helpful starting points for clinicians to consider would be navigating cultural norms of emotional expression, exploring the adaptiveness of Schemas and Modes in different cultural contexts, and culturally suitable ways of addressing Parent/Critic Modes.

## Data Availability

Raw interview data will not be made available to the public to maintain participant confidentiality.

## Appendix

### Interview Schedule

1. What has been your experience using Schema Therapy with your clients?
2. In your experience, has the Schema Therapy model been culturally applicable to your clients? Why/Why not?
3. Do you think Schema Therapy techniques are applicable across cultures? Why/Why not?
4. What have your experiences been, if any, of using Schema Therapy with cultural-based concerns (e.g., acculturation and racism)?
5. How, if at all, has your own cultural context influenced how you practise Schema Therapy?
6. What resources do you think would be helpful for practicing Schema Therapy with clients across cultures?
7. Is there anything else you would like to add about your perspective on culture and Schema Therapy?

## References

[cit0001] Arntz, A., Rijkeboer, M., Chan, E., Fassbinder, E., Karaosmanoglu, A., Lee, C. W., & Panzeri, M. (2021). Towards a reformulated theory underlying schema therapy: Position paper of an international workgroup. *Cognitive Therapy and Research*, 45(6), 1007–15. 10.1007/s10608-021-10209-5

[cit0002] Barbieri, A., Visco-Comandini, F., Trianni, A., & Saliani, A. M. (2022). A schema therapy approach to complex dissociative disorder in a cross-cultural setting: A single case study. *Rivista di psichiatria*, 57(3), 141–157. 10.1708/3814.37993 35695685

[cit0003] Bengtsson, J., Nordin, S., & Carlbring, P. (2015). Therapists’ experiences of conducting cognitive behavioural therapy online vis-à-vis face-to-face. *Cognitive Behaviour Therapy*, 44(6), 470–479. 10.1080/16506073.2015.1053408 26090947

[cit0004] Benuto, L. T., Newlands, R., Singer, J., Casas, J., & Cummings, C. (2021). Culturally sensitive clinical practices: A mixed methods study. *Psychological Services*, 18(4), 632–642. 10.1037/ser0000493 32673036

[cit0005] Bernal, G., Jiménez-Chafey, M. I., & Domenech Rodríguez, M. M. (2009). Cultural adaptation of treatments: A resource for considering culture in evidence-based practice. *Professional Psychology, Research and Practice*, 40(4), 361–368. 10.1037/a0016401

[cit0006] Braun, V., & Clarke, V. (2022). *Thematic analysis: A practical guide*. Sage.

[cit0007] Cabassa, L. J., & Baumann, A. A. (2013). A two-way street: Bridging implementation science and cultural adaptations of mental health treatments. *Implementation Science*, 8(1), 90. 10.1186/1748-5908-8-90 23958445 PMC3765289

[cit0008] Crotty, M. (1998). *Foundations of social research: Meaning and perspective in the research process*. Sage Publications.

[cit0009] Dinh, K. T., & Kalaja, A. (2023). A qualitative examination of cultural influences in parent–child relationships and life satisfaction among Asian American young adults. *Asian American Journal of Psychology*, Advance online publication. 15(2), 83–95. 10.1037/aap0000331

[cit0010] Edge, D., & Lemetyinen, H. (2019). Psychology across cultures: Challenges and opportunities. *Psychology and Psychotherapy*, 92(2), 261–276. 10.1111/papt.12229 31001925

[cit0011] Fonseca, R. G., Marques, P. I. S., da Costa, F. F., Landeira-Fernandez, J., Rimes, K. A., & Mograbi, D. C. (2023). Cross-cultural differences in beliefs about emotions: A comparison between Brazilian and British participants. *Jornal brasileiro de psiquiatria*, 72(3), 152–158. 10.1590/0047-2085000000423

[cit0012] Gallardo, M. E., Johnson, J., Parham, T. A., & Carter, J. A. (2009). Ethics and multiculturalism: Advancing cultural and clinical responsiveness. *Professional Psychology, Research and Practice*, 40(5), 425–435. 10.1037/a0016871

[cit0013] Gülüm, İ. V., & Soygüt, G. (2022). Limited reparenting as a corrective emotional experience in schema therapy: A preliminary task analysis. *Psychotherapy Research*, 32(2), 263–276. 10.1080/10503307.2021.1921301 33910484

[cit0014] Hays, P. A. (2009). Integrating evidence-based practice, cognitive–behavior therapy, and multicultural therapy: Ten steps for culturally competent practice. *Professional Psychology, Research and Practice*, 40(4), 354–360. 10.1037/a0016250

[cit0015] He, Z., Muhlert, N., & Elliott, R. (2021). Emotion regulation of social exclusion: A cross-cultural study. *Humanities and Social Sciences Communications*, 8(1), 173. 10.1057/s41599-021-00857-z

[cit0016] Heath, G., & Startup, H. (2020). *Creative methods in schema therapy: Advances and innovation in clinical practice* (1st ed.). Routledge. 10.4324/9781351171847

[cit0017] Henry, H. M., & Nasreldin, A. (2024). Integrating schema therapy with Kleinman’s cultural explanatory Model: A case study. *Psychological Studies*, 69(1), 105–111. 10.1007/s12646-023-00771-1

[cit0018] Hofstede, G. (2011). Dimensionalising culture: The Hofstede model in context. *Online Readings in Psychology and Culture*, 2(1), 1–12. 10.9707/2307-0919.1014

[cit0019] Huisy-Howland, G. (2021). *Schema connection to Chinese culture*. https://www.schematherapysociety.org/resources/Documents/Bulletin21_Mar21_FINAL.pdf?fbclid=IwAR1dltEc2eg7gFiP9mIXxMqtiAE_M1eqKjfFlpGrdk5EyUIfXl-EWqND4w

[cit0020] International Society of Schema Therapy. (2019). *Approved schema therapy training programs*. https://schematherapysociety.org/Training-Programs

[cit0021] Josek, A. K., Schaich, A., Braakmann, D., Assmann, N., Jauch-Chara, K., Arntz, A., Schweiger, U., & Fassbinder, E. (2023). Chairwork in schema therapy for patients with borderline personality disorder-A qualitative study of patients’ perceptions. *Frontiers in Psychiatry*, 14, 1180839. 10.3389/fpsyt.2023.1180839 37333913 PMC10272534

[cit0022] Joshua, P. R., Lewis, V., Kelty, S. F., & Boer, D. P. (2023). Is schema therapy effective for adults with eating disorders? A systematic review into the evidence. *Cognitive Behaviour Therapy*, 52(3), 213–231. 10.1080/16506073.2022.2158926 36633136

[cit0023] Körük, S., & Özabacı, N. (2018). Effectiveness of schema therapy on the treatment of depressive disorders: A meta-analysis [şema Terapinin Depresif Bozuklukların Tedavisindeki Etkililiği: Bir meta-Analiz]. *Psikiyatride Guncel Yaklasimlar*, 10(4), 460–470. 10.18863/pgy.361790

[cit0024] Lian, A. E. Z., Chooi, W.-T., & Bono, S. A. (2024). The development and the effectiveness of schema therapy on Malaysian female young adults who experienced continuous trauma and post-traumatic stress disorder. *European Journal of Trauma & Dissociation*, 8(3), 100427. 10.1016/j.ejtd.2024.100427

[cit0025] Liddell, B. J., & Williams, E. N. (2019). Cultural differences in interpersonal emotion regulation. *Frontiers in Psychology*, 10, 999. 10.3389/fpsyg.2019.00999 31133934 PMC6523987

[cit0026] Louis, J. P., Wood, A. M., Lockwood, G., Ho, M.-H. R., & Ferguson, E. (2018). Positive clinical psychology and schema therapy (ST): The development of the young positive schema questionnaire (YPSQ) to complement the young schema questionnaire 3 short form (YSQ-S3). *Psychological Assessment*, 30(9), 1199–1213. 10.1037/pas0000567 29672073

[cit0027] Mao, A., Brockman, R., Neo, H. L. M., Siu, S. H. C., Liu, X., & Rhodes, P. (2022). A qualitative inquiry into the acceptability of schema therapy in Hong Kong and Singapore: Implications for cultural responsiveness in the practice of schema therapy. *The Clinical Psychologist*, 26(1), 1–10. 10.1080/13284207.2022.2052273

[cit0028] Marsiglia, F. F., & Booth, J. M. (2015). Cultural adaptation of interventions in real practice settings. *Research on Social Work Practice*, 24(4), 423–432. 10.1177/1049731514535989 PMC4512185 26213454

[cit0029] McSweeney, B. (2002). Hofstede’s model of national cultural differences and their consequences: A triumph of faith - a failure of analysis. *Human Relations*, 55(1), 89–118. 10.1177/0018726702551004

[cit0030] Naeem, F., Phiri, P., Rathod, S., & Ayub, M. (2019). Cultural adaptation of cognitive–behavioural therapy. *BJPsych Advances*, 25(6), 387–395. 10.1192/bja.2019.15

[cit0031] Oettingen, J., Chodkiewicz, J., Macik, D., & Gruszczynska, E. (2018). Polish adaptation of the young schema questionnaire 3 short form (YSQ-S3-PL). *Psychiatria Polska*, 52(4), 707–718. 10.12740/PP/OnlineFirst/76541 30368540

[cit0032] Parsonson, K. L. (2021). *Handbook of international psychology ethics: Codes and commentary from around the world* (1 ed.). Routledge. 10.4324/9780367814250

[cit0033] Peeters, N., van Passel, B., & Krans, J. (2021). The effectiveness of schema therapy for patients with anxiety disorders, OCD, or PTSD: A systematic review and research agenda. *British Journal of Clinical Psychology*, 61(3), 579–597. 10.1111/bjc.12324 34296767 PMC9544733

[cit0034] Pilkington, P. D., Spicer, L., & Wilson, M. (2022). Schema therapists' perceptions of the influence of their early maladaptive schemas on therapy. *Psychotherapy Research*, 32(7), 833–846. 10.1080/10503307.2022.2038804 35179087

[cit0035] Rad, M. S., Martingano, A. J., & Ginges, J. (2018). Toward a psychology of Homo sapiens: Making psychological science more representative of the human population. *Proceedings of the National Academy of Sciences of the United States of America*, 115(45), 11401–11405. 10.1073/pnas.1721165115 30397114 PMC6233089

[cit0036] Rathod, S., Gega, L., Degnan, A., Pikard, J., Khan, T., Husain, N., Munshi, T., & Naeem, F. (2018). The current status of culturally adapted mental health interventions: A practice-focused review of meta-analyses. Neuropsychiatric Disease and Treatment, 14(165–178. 10.2147/NDT.S138430 PMC5757988 29379289

[cit0037] Rathod, S., Phiri, P., & Naeem, F. (2019). An evidence-based framework to culturally adapt cognitive behaviour therapy. *The Cognitive Behaviour Therapist*, 12, e10, Article e10. 10.1017/S1754470X18000247

[cit0038] Ridley, C. R., Sahu, A., Console, K., Surya, S., Tran, V., Xie, S., & Yin, C. (2021). The process model of multicultural counseling competence. *The Counseling Psychologist*, 49(4), 534–567. 10.1177/0011000021992339

[cit0039] Sakulsriprasert, C., Phukao, D., Kanjanawong, S., & Meemon, N. (2016). The reliability and factor structure of Thai young schema questionnaire-short form 3. *Asian Journal of Psychiatry*, 24, 85–90. 10.1016/j.ajp.2016.09.011 27931916

[cit0040] Soheilian, S. S., Inman, A. G., Klinger, R. S., Isenberg, D. S., & Kulp, L. E. (2014). Multicultural supervision: Supervisees’ reflections on culturally competent supervision. *Counselling Psychology Quarterly*, 27(4), 379–392. 10.1080/09515070.2014.961408

[cit0041] Sorenson, C., & Harrell, S. P. (2021). Development and testing of the 4-Domain cultural adaptation Model (CAM4). *Professional Psychology, Research and Practice*, 52(3), 250–259. 10.1037/pro0000370

[cit0042] Wang, S.-W., & Lau, A. S. (2015). Mutual and non-mutual social support: Cultural differences in the psychological, behavioral, and biological effects of support seeking. *Journal of Cross-Cultural Psychology*, 46(7), 916–929. 10.1177/0022022115592967

[cit0043] Young, J. E. (2005). *Young schema questionnaire - short form 3 (YSQ-S3)*. Cognitive Therapy Center.

[cit0044] Young, J. E., Klosko, J. S., & Weishaar, M. E. (2003). *Schema therapy: A practitioner’s guide*. The Guilford Press.

[cit0045] Zhang, K., Hu, X., Ma, L., Xie, Q., Wang, Z., Fan, C., & Li, X. (2023). The efficacy of schema therapy for personality disorders: A systematic review and meta-analysis. *Nordic Journal of Psychiatry*, 77(7), 641–650. 10.1080/08039488.2023.2228304 37402124

